# Supplementary material for: Dyslipidemia is a metabolic hallmark of acute pain in sickle cell disease
Source: medRxiv. 2026 Jun 29:2026.06.24.26356495. Preprint. [Version 1] doi: 10.64898/2026.06.24.26356495 (PMC13345495; doi:10.64898/2026.06.24.26356495)

medRxiv preprint doi: https://doi.org/10.64898/2026.06.24.26356495; this version posted June 29, 2026. The copyright holder for this preprint (which was not certified by peer review) is the author/funder, who has granted medRxiv a license to display the preprint in perpetuity.
It is made available under a CC-BY 4.0 International license .

Supplemental Figures

S. Figure 1. Tricarboxylic acid metabolites are dysregulated during acute pain in SCD.

Individual tricarboxylic acid metabolites are decreased in plasma from children and adolescents with SCD during acute pain compared to SCD baseline health.

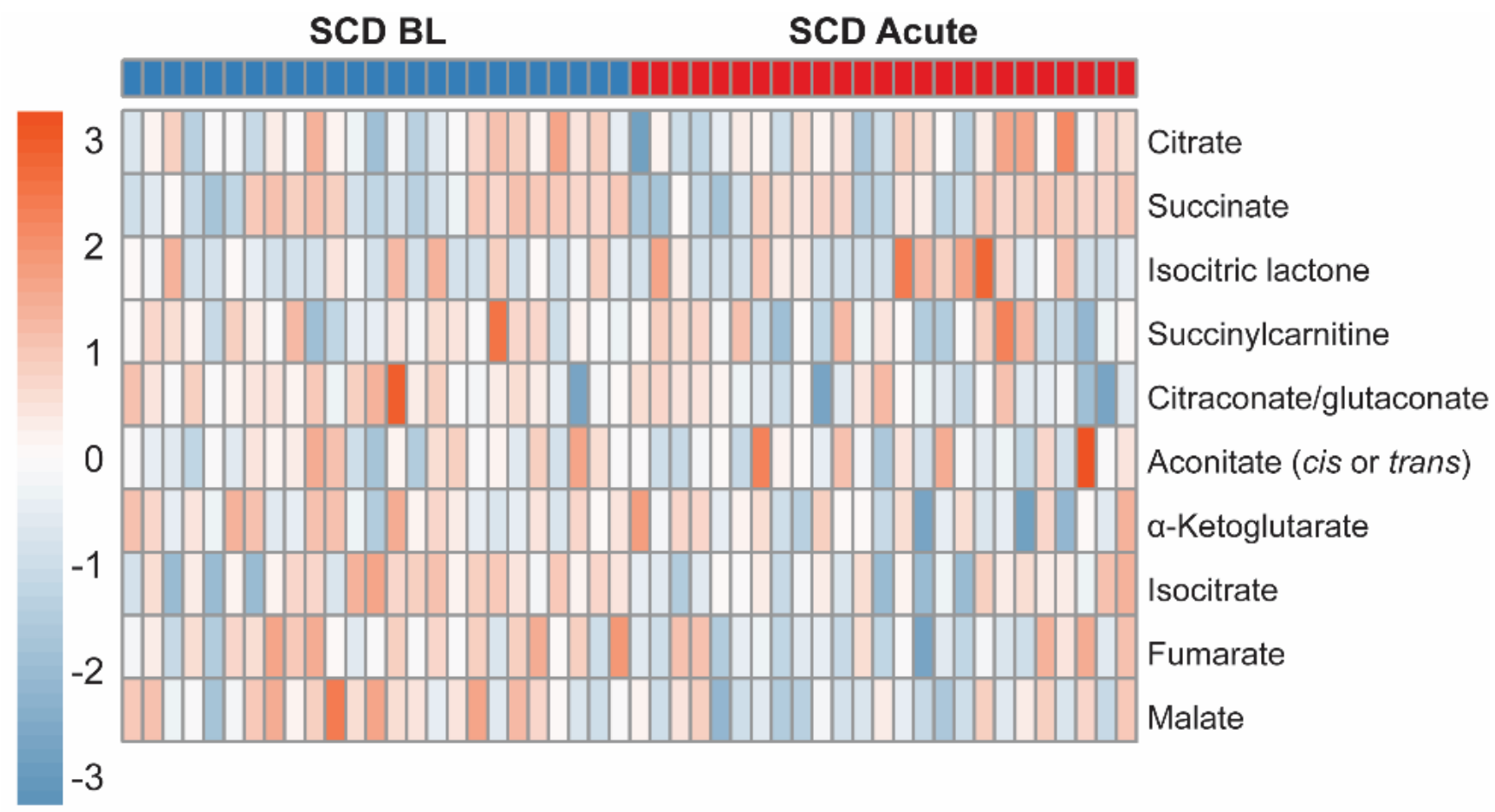

S. Figure 2. Porphyrin metabolites are increased during baseline health in SCD. (*A*) Metabolites associated with porphyrin metabolism are likewise elevated during baseline health in SCD, but not further elevated during acute pain (*B*).

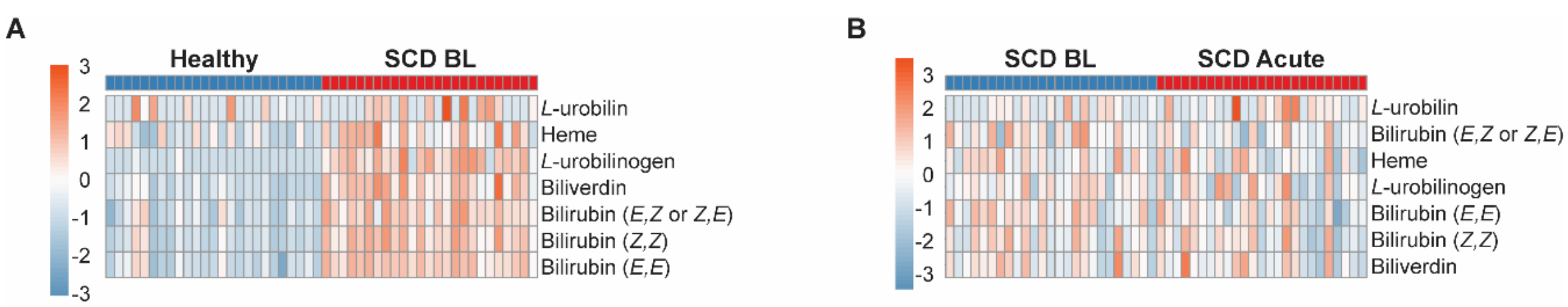

Supplement: Supplementary file 1 [file NIHPP2026.06.24.26356495V1-supplement-1.pdf]
